# Health Checkup and Telemedical Intervention Program for Preventive Medicine in Developing Countries: Verification Study

**DOI:** 10.2196/jmir.3705

**Published:** 2015-01-28

**Authors:** Yasunobu Nohara, Eiko Kai, Partha Pratim Ghosh, Rafiqul Islam, Ashir Ahmed, Masahiro Kuroda, Sozo Inoue, Tatsuo Hiramatsu, Michio Kimura, Shuji Shimizu, Kunihisa Kobayashi, Yukino Baba, Hisashi Kashima, Koji Tsuda, Masashi Sugiyama, Mathieu Blondel, Naonori Ueda, Masaru Kitsuregawa, Naoki Nakashima

**Affiliations:** ^1^Medical Information CenterKyushu University HospitalFukuokaJapan; ^2^Graduate School of Information Science and Electrical EngineeringKyushu UniversityFukuokaJapan; ^3^Grameen CommunicationsDhakaBangladesh; ^4^National Institute of Information and Communications TechnologyKoganei, TokyoJapan; ^5^Faculty of EngineeringKyushu Institute of TechnologyKitakyushuJapan; ^6^Graduate School of MedicineThe University of TokyoTokyoJapan; ^7^Department of Medical InformaticsHamamatsu University School of MedicineHamamatsuJapan; ^8^Department of Endoscopic Diagnostics TherapeuticsKyushu University HospitalFukuokaJapan; ^9^Department of Endocrinology and Diabetes MellitusFukuoka University Chikushi HospitalChikushinoJapan; ^10^National Institute of InformaticsTokyoJapan; ^11^Graduate School of InformaticsKyoto UniversityKyotoJapan; ^12^Graduate School of Frontier SciencesThe University of TokyoKashiwaJapan; ^13^Graduate School of Information Science and EngineeringTokyo Institute of TechnologyTokyoJapan; ^14^NTT Communication Science LaboratoriesKyotoJapan; ^15^Institute of Industrial ScienceThe University of TokyoTokyoJapan

**Keywords:** public health informatics, preventive medicine, teleconsultation, body area network, sensor, developing countries

## Abstract

**Background:**

The prevalence of non-communicable diseases is increasing throughout the world, including developing countries.

**Objective:**

The intent was to conduct a study of a preventive medical service in a developing country, combining eHealth checkups and teleconsultation as well as assess stratification rules and the short-term effects of intervention.

**Methods:**

We developed an eHealth system that comprises a set of sensor devices in an attaché case, a data transmission system linked to a mobile network, and a data management application. We provided eHealth checkups for the populations of five villages and the employees of five factories/offices in Bangladesh. Individual health condition was automatically categorized into four grades based on international diagnostic standards: green (healthy), yellow (caution), orange (affected), and red (emergent). We provided teleconsultation for orange- and red-grade subjects and we provided teleprescription for these subjects as required.

**Results:**

The first checkup was provided to 16,741 subjects. After one year, 2361 subjects participated in the second checkup and the systolic blood pressure of these subjects was significantly decreased from an average of 121 mmHg to an average of 116 mmHg (*P*<.001). Based on these results, we propose a cost-effective method using a machine learning technique (random forest method) using the medical interview, subject profiles, and checkup results as predictor to avoid costly measurements of blood sugar, to ensure sustainability of the program in developing countries.

**Conclusions:**

The results of this study demonstrate the benefits of an eHealth checkup and teleconsultation program as an effective health care system in developing countries.

##  Introduction

The prevalence of non-communicable diseases (NCDs), such as heart disease, stroke, cancer, chronic kidney diseases, and diabetes mellitus, has been increasing rapidly worldwide. The World Health Organization reported that NCDs accounted for 63% (36 million) of the 57 million global deaths in 2008 and approximately 80% of all NCD-related deaths occurred in low- and middle-income countries [1]. In these developing countries, 29% of NCD-related deaths occurred in the working-age group (in people aged <60 years). This rate is higher than that for high-income countries (13%) and contributes to declining labor productivity in developing countries. The total number of annual NCD-related deaths is estimated to reach 55 million by 2030 [2]. NCDs are no longer just a problem for high-income countries, but a problem that affects all countries.

Preventive medicine is the key to combat NCDs. Preventive medicine comprises three levels: primary prevention (maintaining a healthy condition), secondary prevention (avoiding the development of NCDs), and tertiary prevention (preventing the progression of NCDs into serious medical conditions). Over time, the focus of medical services in developed countries has changed from acute and serious diseases to the management of chronic diseases. Developing countries have the opportunity to follow a different path, through the implementation of low-cost preventive and compassionate health care/medical services based on information and communication technology (ICT) [3].

In this study, we aimed to evaluate the impact of our preventive health care/medical program consisting of primary, secondary, and tertiary prevention services provided to >10,000 subjects in Bangladesh. We selected Bangladesh as the research area because, while there are few medical institutions in rural areas, there are many pharmacies and the mobile Internet network has spread throughout the nation, as is the case in many developing countries.

We conducted the research over 2 years, applying eHealth solutions and telemedical interventions in an attempt to ensure an accurate stratification balance and assess the effects of intervention after 1 year in >2000 subjects.

The target diseases in this program are chronic NCDs including diabetes mellitus and hypertension, which are rapidly increasing in developing countries. Sensor devices, including blood glucose meters and blood pressure meters, have been developed for the management of these diseases and are widely available. The World Bank Group’s *Disease Control Priorities in Developing Countries* has also emphasized the paramount importance of risk management of chronic NCDs [4]. Using machine learning, we attempted to establish a method to decrease the cost of health checkups by predicting the results of expensive health check tests.

## Methods

### Overview

We developed an eHealth system named the Portable Health Clinic (PHC). PHC comprises a set of sensor devices in an attaché case, a data transmission system linked to a mobile network, and a data management application. The system can be used by operators with minimal information technology literacy to provide health checkup services, even in rural areas. We included a teleconsultation service using Skype over the mobile network to gather data on health. To assess the usability and sustainability of the system, we designed a study model including local pharmacies to provide a teleprescription service. We conducted a field study from July 2012 to March 2014 (first year: July 2012-February 2013; second year: June 2013-March 2014).

### The Portable Health Clinic

We selected sensor devices based on international information standards and approved by Japanese pharmaceutical law. If a device did not have a standard transmission format, we attached a body area network (BAN) interface to the sensor. The BAN was published as IEEE802.15.6 in 2012 and uses frequency bands approved by national medical and/or regulatory authorities and the industrial, scientific, and medical (ISM) band [5]. In addition to the dedicated medical bands, it provides quality service, extremely low power, and a data speed of up to 10 Mbps, supports medical security, and emergency data handling.

**Figure 1:**
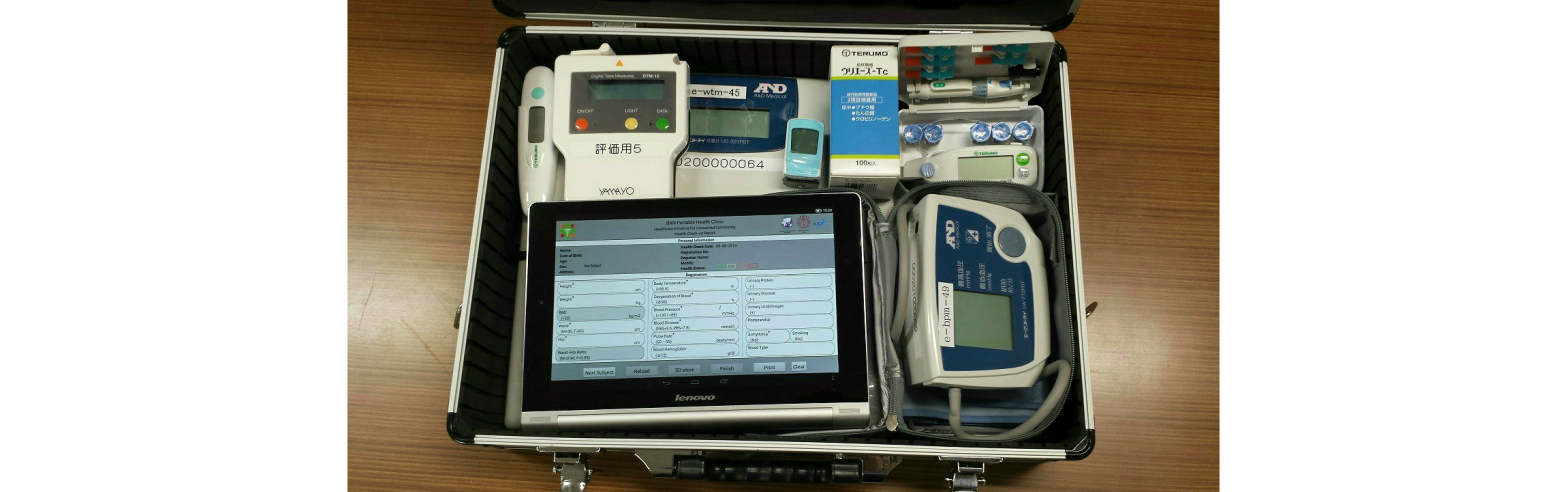
The Portable Health Clinic System package.

For easy portability, we put the components into an attaché case. The attaché case was equipped with an Android tablet, consumable goods, including urine and blood sugar test strips, batteries, paper, and pens. The total weight of the attaché case and contents was approximately 10 kg (Figure 1).

An Android tablet served as a data input terminal, aggregating data via BAN and manual input and communicating with the sensor server. Results of individual health checkups, including the stratification level, were shown on a local site. The main server in a medical call center in Dhaka, the capital of Bangladesh, stored all sensor data. Data was available to doctors through the call center.

Local servers temporarily stored data from the Android tablet via wireless-LAN and synchronized data with the main server when an Internet connection was available. This use of local servers enabled PHC operators to upload their data even if an Internet connection was temporarily unavailable.

### Stratification Algorithm

Before the study commenced, we established “Bangladesh-logic” for risk stratification using international diagnostic standards [6-10] to rank the risk grade into four groups—green (healthy), yellow (caution), orange (affected), and red (emergent)—based on the results of each health checkup item (Table 1). The overall health condition of each subject was also determined by integrating the results of questionnaires into the four groups by the worst color of all health checkup items. Examples of the determination of overall health conditions based on the results of each health checkup item are as follows:

- Green, Orange, Green, …, Yellow → Orange
- Green, Green, …, Green (all Green) → Green

The presence or absence of arrhythmia was determined using a blood pressure meter. Data on smoking and time since the last meal were obtained from questionnaires.

**Table 1:** Bangladesh-logic: criteria for risk stratification.

		Green	Yellow	Orange	Red
**Waist (cm)**					
	Male	<90	≥90		
	Female	<80	≥80		
**Waist/hip ratio**					
	Male	<0.90	≥0.90		
	Female	<0.85	≥0.85		
Body mass index (kg/m^2^)		<25	≥25, <30	≥30, <35	≥35
**Blood pressure (mmHg)**					
	Systolic	<130	≥130, <140	≥140, <180	≥180
	Diastolic	<85	≥85, <90	≥90, <110	≥110
**Blood sugar (mg/dl)**					
	Fasting	<100	≥100, <126	≥126, <200	≥200
	Postprandial	<140	≥140, <200	≥200, <300	≥300
Urine-protein		Negative	Trace	Positive	
Urine-sugar		Negative	Trace	Positive	
Urine-urobilinogen		Trace		Positive	
Pulse rate (bpm)		≥60, <100	≥50, <60 ≥100, <120	<50 ≥120	
Arrhythmia		No		Yes	
Smoking		None	+		
Body temperature (°C)		<37	≥37, <37.5	≥37.5	
Oxygen saturation (SpO_2_) (%)		≥96	≥93, <96	≥90, <93	<90

### Questionnaires on First and Second Visits

During health checkup visits, we surveyed subjects using questionnaires written in Bengali. Because the literacy rate is <60% in Bangladesh, a staff member read the questionnaire to the subjects and entered response data into the system. On the first visit, we asked about literacy, occupation, time since the last meal, present symptoms, past diseases, medication, smoking, weight change, exercise, walking speed, eating behavior, sleeping, and the desire to have a healthy lifestyle. For orange- and red-grade subjects, we administered the questionnaire for teleconsultation with questions including information on drug allergy and surgical history. For subjects who participated in both the first and second years, we administered a different questionnaire at the second visit with questions regarding memory and effects of the first health checkup, psychological (Prochaska’s) staging, present symptoms, and medication.

### System Operation

**Figure 2:**
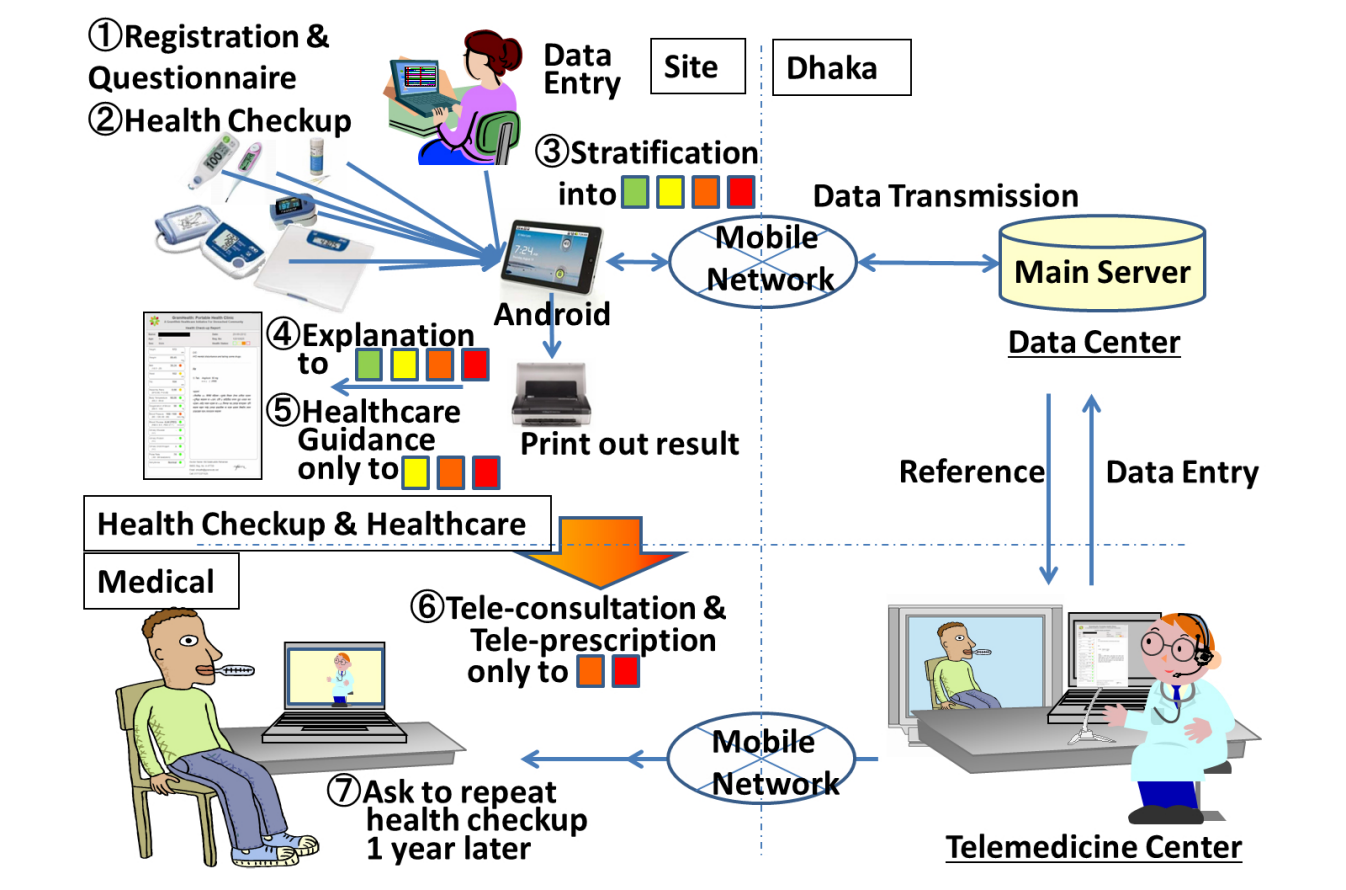
Work flow (Steps 1–7) and data flow (arrows) of the service.

We provided a health care service for the study, including a health checkup using sensor devices in the PHC, data storage in the call center, a health report, health care guidance according to the situation of individuals, and a teleconsultation with a doctor in the medical call center (Figure 2). We conducted the study in five rural villages and five factories/offices in Bangladesh. In the first year, we conducted the study around Dhaka (Dhaka, Shariatpur, Chandpur, and Gazipur) because of a logistics problem. In the second year, we selected sites from all over the country to check the country's health status. The sites of the second year are Chittagong (south-eastern area), Rajshahi (western area), Thakurgaon (north-western area), and around Dhaka.

At the first visit, after registration, the subjects received an ID card with a barcode (Step 1 in Figure 2). After completing the questionnaire, the subject underwent the health checkup with the sensor devices in the PHC (Step 2). Both the blood glucose and urine tests were performed by qualified health care professionals, whereas the other tests were performed by trained staff. We cross-checked the urine test results of the workers every 2 or 3 months because this test requires visual judgment. Other devices, including blood glucose devices, display numerical results and we do not require calibration among workers. The data were stored in an Android tablet and in the main server in Dhaka. Categorized results for the four risk groups, graded from green to red according to the Bangladesh-logic, were printed out (Steps 3 and 4) and explained to the subject by the local staff (Step 4). A booklet was provided to all subjects graded yellow, orange, or red (Step 5). We provided telemedical intervention with a doctor in Dhaka for orange- and red-grade subjects (Step 6). Because we selected sites from around Dhaka in the first year, subjects in the village and factories/offices around Dhaka were asked to undergo the health checkup 1 year later to enable assessment of the effects of the program (Step 7).

### Teleconsultation and Teleprescription

After the health checkup, we provided telemedical intervention for orange- and red-grade subjects via mobile network contact (Skype) with the medical call center in Dhaka. Because most areas in Bangladesh have Internet access (2G/3G), we brought laptop PCs or tablet PCs (iPad) with mobile routers to the checkup sites. The staff set up special rooms for teleconsultations at checkup sites and assisted subjects to communicate with remote doctors in Dhaka. Doctors had access to the results of health checkups via the Internet and they were able to provide advice on disease management and encourage subjects to visit a clinic. Where required, the doctors could send a teleprescription for anti-hypertensive medication via the network. In our program, subjects who received a teleprescription could visit their local pharmacy to purchase medication.

### Booklet for Health Guidance

We provided an 11-page booklet to educate subjects graded yellow, orange, or red. The booklet contained information on the risks of NCDs, including obesity, hypertension, diabetes mellitus, smoking-related diseases, and chronic kidney disease. We prepared both English and Bengali versions of the booklet and provided the Bengali version to the subjects in the study. For subjects who could not read Bengali, a staff member explained the checkup results and provided health care guidance orally.

### Ethical Considerations

The Kyushu University Institutional Review Board for Clinical Trials approved the protocol of this verification study in 2012. We applied for the IRB of Kyushu University, Japan, because participant groups in Bangladesh had no IRB. We prepared a consent form, etc, after discussing with the local doctors.

## Results

### Overview

**Figure 3:**
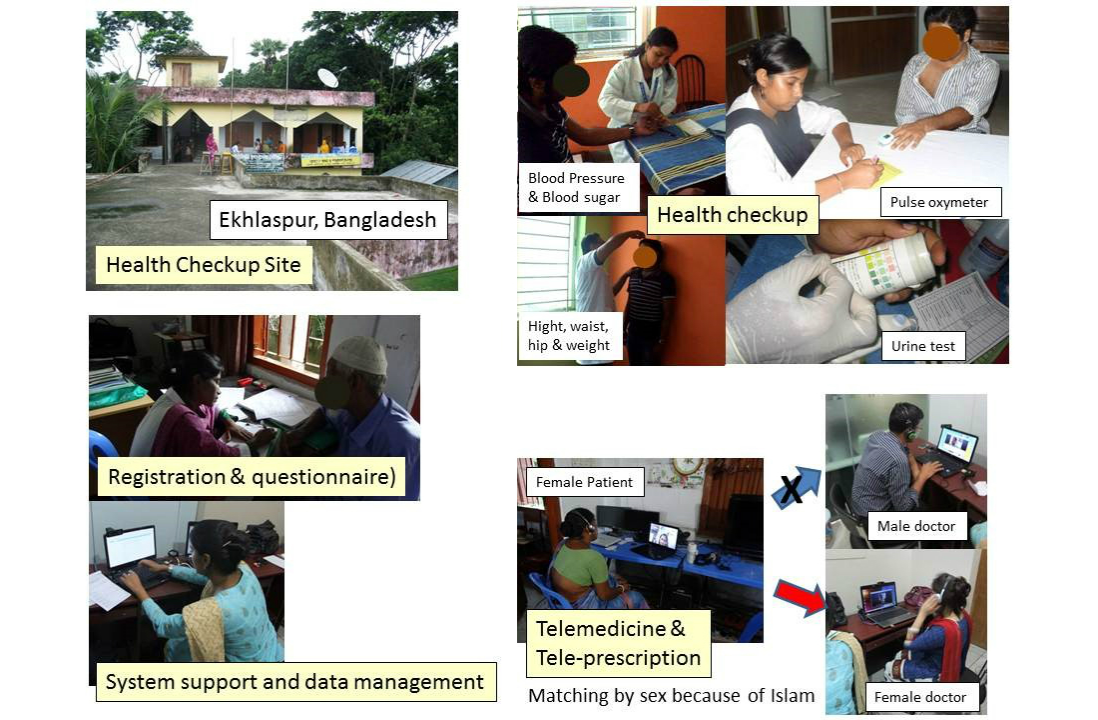
Images of a health checkup and teleconsultation.

There were 16,741 subjects assessed at the first health checkup, 9143 (54.61%) males and 7598 (45.39%) females (Table 2). There were 9309 (55.61%) subjects from urban areas and 7432 (44.39%) subjects from rural areas. Most of the subjects in urban areas were male (male/female=6299/3010), whereas female subjects were more numerous in the rural areas (male/female=2844/4588). Figure 3 shows images of the health checkup and teleconsultation process in a rural area.

**Figure 4:**
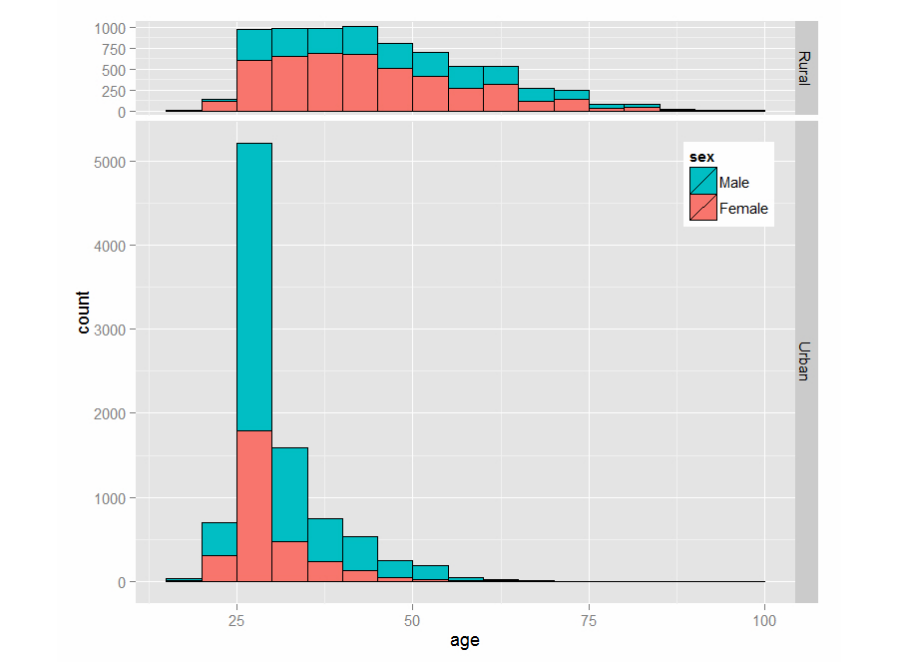
Age distribution for rural and urban areas.

Figure 4 shows the age distribution for rural and urban areas. There was a wide age distribution in rural areas, while there was a clear peak in the number of subjects aged in the late 20s in urban areas. The average age was 35.1 (SD 12.7) years for male subjects and 36.7 (SD 12.8) years for female subjects. The average age was 43.6 (SD 14.0) years for rural subjects and 29.6 (SD 6.9) years for urban subjects.

**Figure 5:**
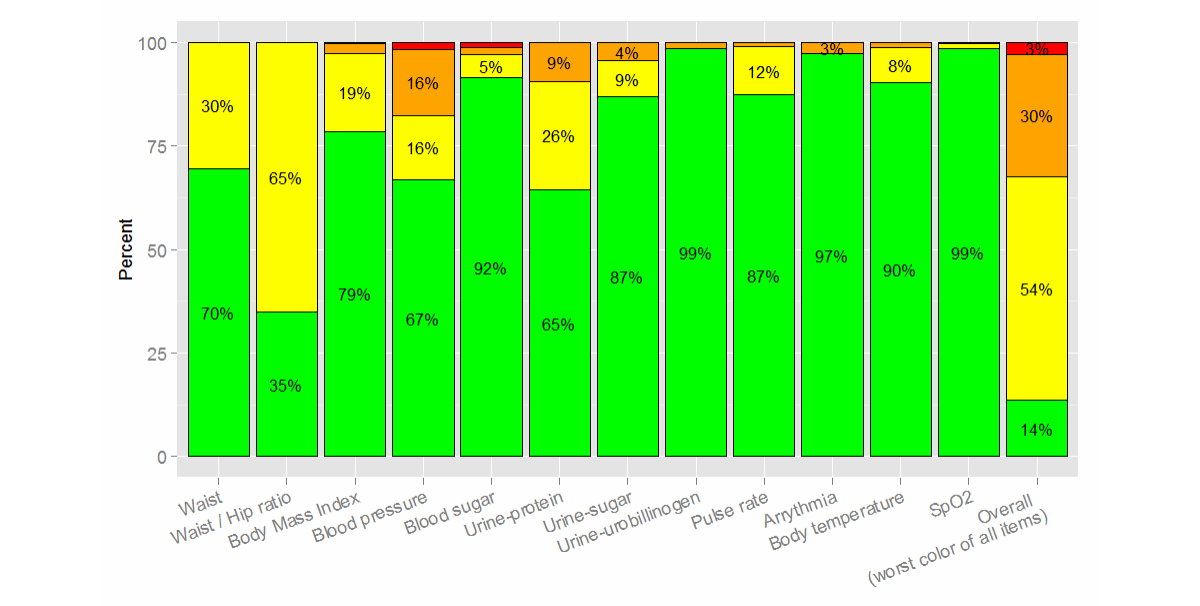
Detailed results showing grading (green, yellow, orange, or red) for each investigation.

The results of the first health checkup are shown in Figure 5. Based on the assessment of overall health condition, we identified 5419 out of 16,741 subjects (32.37%) as affected (orange or red) and 9057 subjects (54.10%) as caution required (yellow). There were 10,879 subjects (64.98% of the total 16,741) graded yellow based on the waist/hip ratio, 5535 (33.06%) graded yellow or higher based on a blood pressure test, and 1402 (8.37%) graded yellow or higher based on a blood sugar test. Subjects were graded red (emergent) based on body mass index (BMI) (39 subjects), blood pressure (258), blood glucose (181), and oxygen saturation (SpO_2_; 6). We provided a teleconsultation service to affected subjects (orange or red, n=4899).

**Table 2:** The number of subjects by sex and location (the number of subjects who participated in both the 2012 and 2013 checkups are indicated in parentheses).

Location	Male	Female	Total
Rural	2844 (177)	4588 (234)	7432 (411)
Urban	6299 (1412)	3010 (538)	9309 (1950)
Total	9143 (1589)	7598 (772)	16,741 (2361)

### Risk Factors Associated with Overall Health Condition

**Figure 6:**
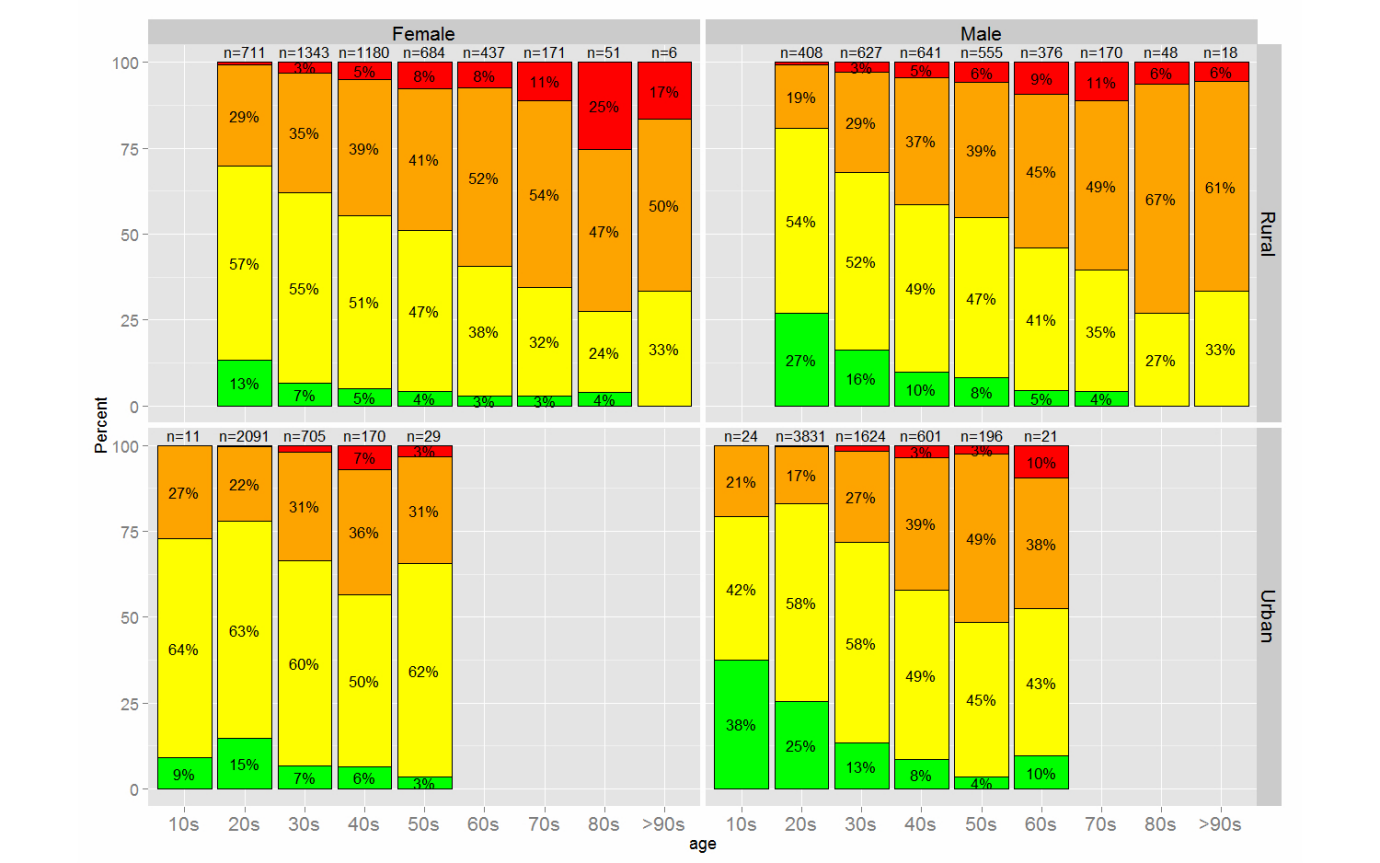
Overall results by age, sex, and area (subject numbers are shown above the columns).

Figure 6 shows the overall results with regard to age, sex, and area. To identify risk factors for NCDs related to overall health condition results, we used logistic regression analysis with the overall result (orange/red: true or green/yellow: false) as the outcome and age, sex, site type, occupation, and literacy as independent variables (n=16,315). The results are presented in Table 3. Variables that were significantly associated with overall health condition (*P*<.05) are noted.

The results of the analysis indicate that older age, female, and living in a rural area were risk factors for NCDs. Contrary to our expectations, literacy (and not illiteracy) was also a risk factor. When we changed the outcome of logistic regression analysis from the overall result to the result of each individual checkup item, we found that literacy was also a risk factor for high BMI, blood pressure, blood glucose, and urine glucose. Conversely, there was no significant difference between literacy and illiteracy for urine protein and urobilinogen levels, pulse rate, arrhythmia, body temperature, and SpO_2_.

Because significant variables were related to body mass, we generated the hypothesis that literate subjects: (1) earn more and tend to overeat, (2) do not get enough exercise because they use their own mode of transport or public transport, and (3) lack a basic awareness of health.

**Table 3:** Risk factors for NCDs associated with the overall health.

Variables		Odds ratio (95% CI)	*P* value
Age		1.04 (1.04-1.05)	<.001^a^
Sex (male)		0.78 (0.71-0.86)	<.001^a^
Area (urban)		0.66 (0.56-0.78)	<.001^a^
**Occupation**			
	Daily labor	0.56 (0.45-0.69)	<.001^a^
	Business	0.83 (0.68-1.01)	.063
	Private/government service	1.00 (reference)	−
	Student	0.43 (0.27-0.66)	<.001^a^
	Housewife	0.86 (0.71-1.03)	.108
	Unemployed	0.80 (0.64-1.01)	.055
Literacy		1.24 (1.14-1.36)	<.001^a^

^a^Variables were significantly associated with overall health condition (*P*<.05).

### Comparison With Results of Health Checkups in Japan

**Figure 7:**
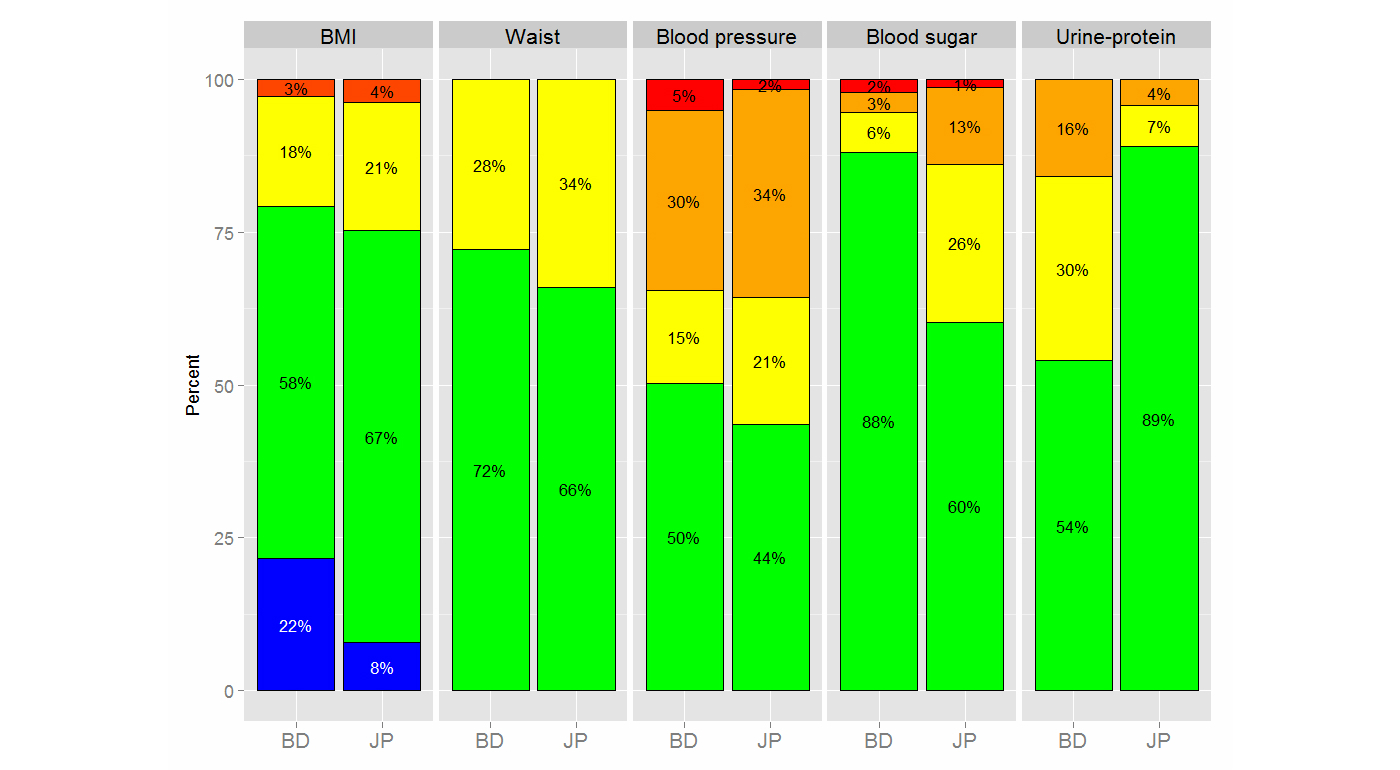
Comparison of health checkup results for Bangladeshi (BD) and Japanese (JP) subjects.

Because NCD problems are spreading from advanced countries to developing countries, it is important for preventive medicine in developing countries to use past experiences in advanced countries. Moreover, experiences in developing countries could improve preventive medicine in advanced countries, the so-called “reverse innovation”. On the other hand, problems specific to each country exist and we need to cope with individual issues. In order to separate the problems, we compared the results of the present study with data from the 2012 National Health and Nutrition Survey in Japan (n>15,000), which was conducted by the Japanese Ministry of Health, Labor and Welfare [11]. The results of the present study were corrected to match the sex and age distribution of the Japanese dataset. Figure 7 shows the results of the comparison. The blue color in the BMI column indicates BMI <18.5 kg/m^2^, meaning subjects were underweight.

**Figure 8:**
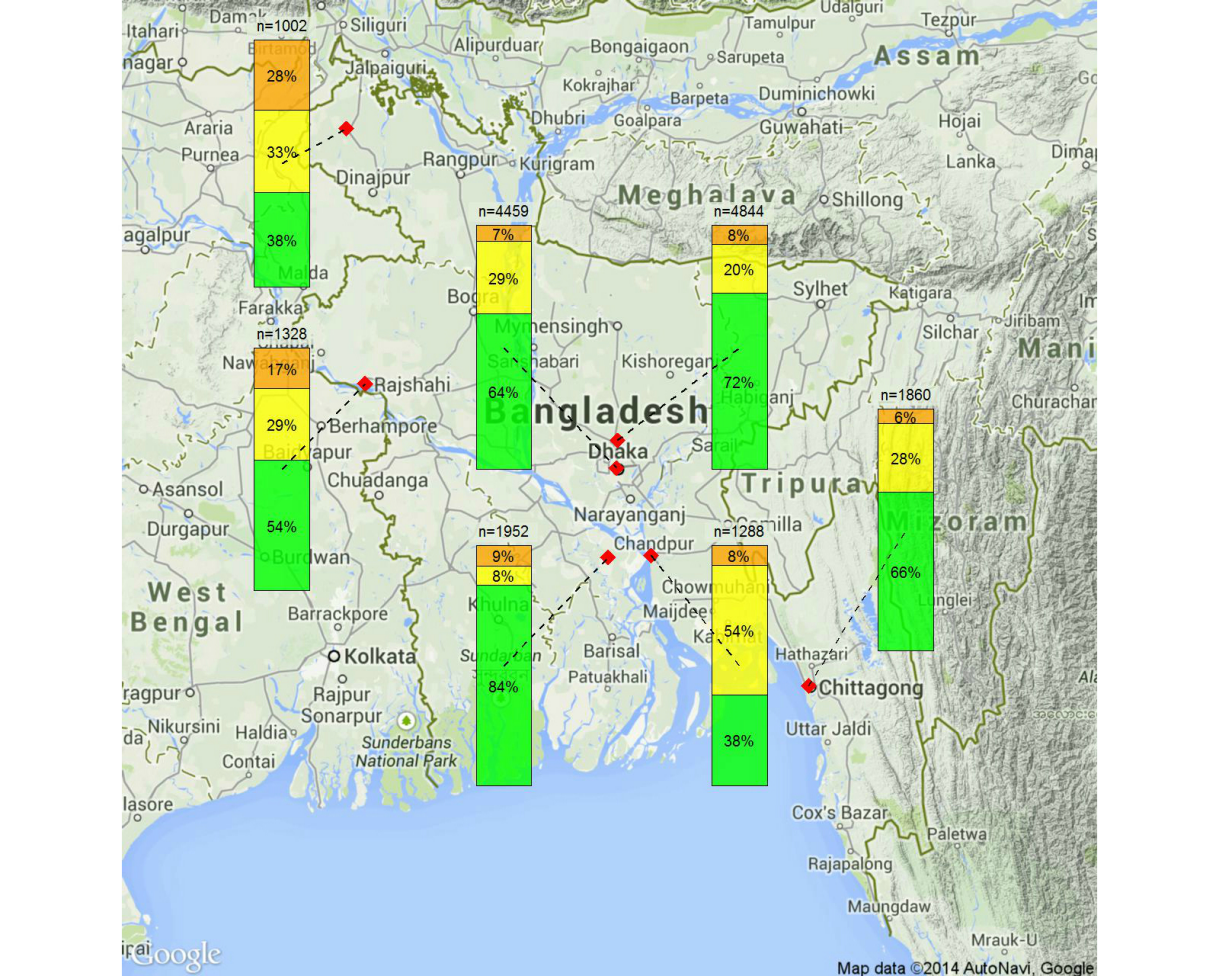
Results of the urine protein test for each checkup site in Bangladesh.

The comparison shows that there were many underweight subjects (blue in BMI) in Bangladesh and that more Bangladeshi subjects were ranked green compared with Japanese subjects. However, the number of Bangladeshi and Japanese subjects ranked orange and red for BMI, waist/hip ratio, and blood pressure was similar, despite very different average income, living conditions, and eating habits in the two countries. Conversely, the results of blood sugar and urine protein tests were quite different between the two countries. This may be a result of regional differences because the results of the urine protein test differed among sites in Bangladesh (Figure 8). We would like conduct further research and learn to cope with the individual issues in Bangladesh.

### The Second Health Checkup

**Figure 9:**
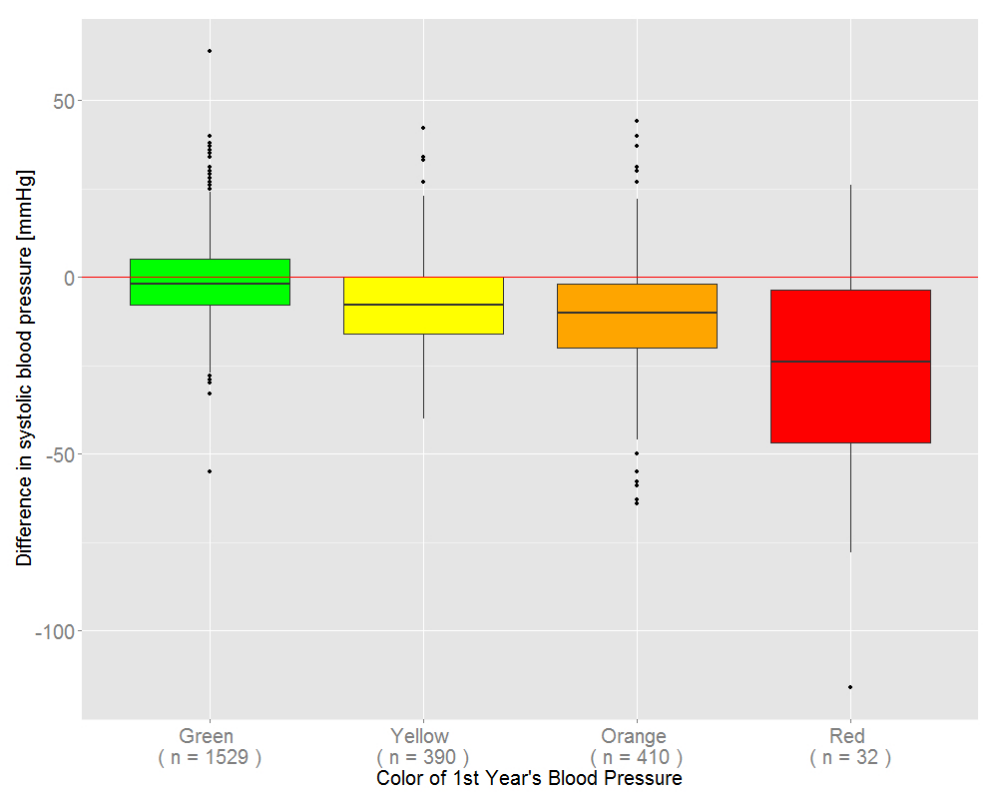
Difference in systolic blood pressure based on color ranking at the first checkup.

There were 2361 subjects who participated in both the 2012 and 2013 health checkups. The details are indicated in parentheses in Table 2. Mean systolic blood pressure (SBP) in the first year (2012) was 121 (SD 17) mmHg and in 2013 it was 116 (SD 15) mmHg. Figure 9 shows the difference in SBP between the 2 years arranged by the ranked color of the first blood pressure test. There was a significant decrease in SBP for all color rankings (*P*<.001).

To determine which subjects showed improved health over the study period, we attempted to predict which subjects would have better health at the second checkup. There were 2110 subjects who had a medical interview at the first checkup and 640 of those subjects were graded as red or orange. Of those 640 subjects, 326 improved their health to green or yellow in the second year.

To further investigate the subjects with improved health, we applied a machine learning technique, the random forest method [12], using the medical interview, subject profiles, and checkup results as the explanatory variables. From the 640-subject dataset, we separated 60% as training sets and 40% as test sets without replacement and ran the estimation. The area under curve (AUC) for 20 trials was 0.7676 (SD 0.0267). The main factors that contributed to the estimation were age, BMI, waist/hip ratio, urine protein, and blood pressure. Based on these findings, we proposed a preferred intervention to help subjects to improve their health.

### Predicting Blood Glucose Test Results

**Figure 10:**
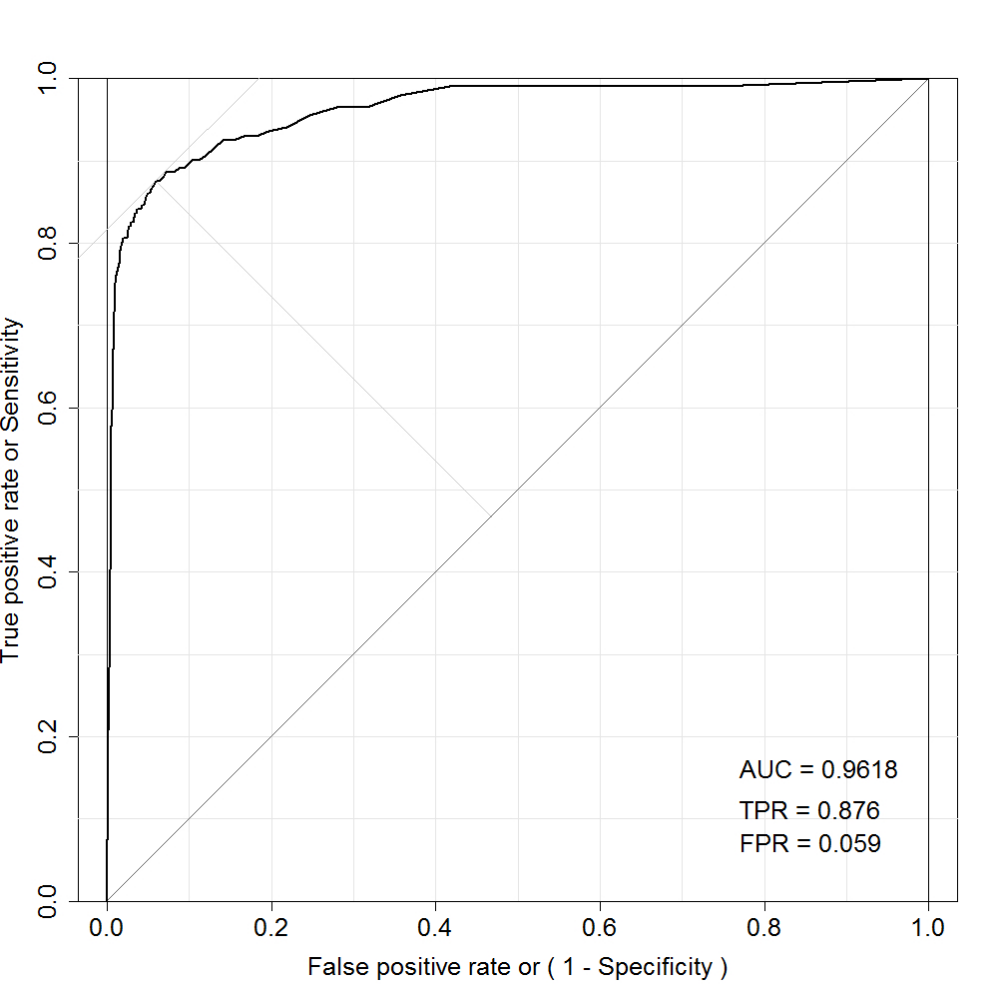
ROC curve for predicting blood sugar.

We applied the random forest method [12] using the medical interview, subject profiles, and checkup results (excluding the blood glucose test result) as explanatory variables to estimate the Bangladesh-logic ranking of red and orange based on the blood glucose test. From the 15,705-subject dataset (true/false=462/15,246), we separated 60% as training sets and 40% as test sets without replacement, and ran the estimation. AUC for 20 trials was 0.9565 (SD 0.0072). Figure 10 shows a receiver operating characteristic (ROC) curve (AUC=0.9618).

Based on Youden’s index, which maximizes the distance from the 45° line on the ROC curve (the upper left point in Figure 10), the true positive rate was 87.6% and the false positive rate was 5.9%.

## Discussion

### Comparison With Prior Work

In recent years, there have been many projects aimed at improving health care in developing countries. Some projects have focused on more specialized and technical approaches, including immunological or enzymatic assays for bacterial toxins [13], and retinal photography, Doppler imaging, biothesiometry, and electrocardiography to detect diabetic complications [14]. Ramachandran et al [15] showed that lifestyle modification could prevent type 2 diabetes in Asian Indian subjects. We targeted the general population and focused on primary prevention; therefore, our eHealth system comprised only basic biosensors to conduct the health checkup.

In this study, we detailed the design of our health care program and presented the results of the study conducted in five villages and five factories/offices in Bangladesh. A study of an Android-based mHealth system in South Africa showed that the system was more cost-effective than pen and paper alternatives [16]. This finding matches our experience in the present study. An intervention program in India found that mobile phone messaging (eg, short messaging service) was an effective and acceptable method for the delivery of advice and support for lifestyle modification to prevent type 2 diabetes in men at high risk [17]. The results of our study suggest that literate subjects are at high risk of NCDs based on high BMI, blood pressure, blood glucose, and urine glucose results, and a mobile phone messaging system would be an effective approach to improving their health.

### Results of the Health Checkup

We found a high rate of obesity based on a high waist/hip ratio (metabolic syndrome) and a high rate of hypertension at the first health checkup. A high carbohydrate and oil-rich diet may contribute to obesity in Bangladesh. In addition, the use of salt to preserve food where refrigeration is not widely available may increase the risk of hypertension. Conversely, despite the high prevalence of obesity, diabetes prevalence was not high, probably a result of high exercise levels. Many subjects were graded yellow based on the urine protein test. Chowdhury et al [18] have reported widespread arsenic contamination of drinking water in Bangladesh. We are currently investigating whether pollution with arsenic and heavy metals, such as cadmium, affects urine tests directly or causes kidney dysfunction.

At the first checkup, 472 of 16,741 subjects were graded red. This is potentially an important outcome of the study because we were able to initiate intervention for these high-risk subjects with health care guidance, teleconsultation, and encouragement to visit a clinic.

At the second checkup, there was a significant decrease in SBP for all color rankings (*P*<.001), even if the subjects were graded as green or yellow at the first checkup. This result indicates that the health of the subjects improved even with knowledge of the initial result and basic health guidance without intervention by a doctor.

### Cost Evaluation

In this study, we performed all the available tests in all subjects. In our estimation, to enable sustainable operation and widespread implementation of the program, we need to reduce the total cost to <US$3 per subject. However, the cost of the blood glucose test is high, at approximately US$0.60 per measurement. We identified effective ways to reduce this cost by estimating the risk for diabetes using predictors and measuring blood glucose only in high-risk subjects.

In designing a predictor system, there has to be a tradeoff between true positive rate (TPR) and false positive rate (FPR) results. For example, we selected a threshold for Youden’s index that minimized the balanced error rate (=1−TPR+FPR), and generated a predictor with a TPR of 87.6% and a FPR of 5.9%. That result indicates that we can skip 14,344 (15,243×94.1%) unnecessary tests if we accept 57 (462×12.4%) oversights. The predictor system would reduce the number of blood glucose tests from 15,705 to 1304; consequently, the measurement cost per subject would be reduced to one-tenth.

We need to design a predictor that maximizes TPR under existing budget constraints, to manage the health of a large group with acceptable FPRs. To be more precise, if we arrange the risk value in the descending order first and count the number by the limits of inspection, then we can choose the risk value as the threshold.

The cost of teleconsultation is also high because of the high salary paid to doctors. Reducing the workload of doctors reduces the cost of the medical program. We are currently analyzing eHealth records using the association rule to support the clinical decisions of medical staff [19]. This analysis can help doctors to add prescription data into the system faster because the system predicts what the doctors want to do and can show candidate inputs and instructions. Machine learning techniques could substitute formulaic, insignificant, and cumbersome work of doctors, enabling them to concentrate on more specific and important issues of patients.

In this study, we provided a health guidance booklet for subjects and a staff member explained the checkup results and health care guidelines orally for illiterate subjects. Because cost control is a serious concern of this project, we plan to make educational videos for health guidance and screen them on devices such as tablet PCs, etc, at the checkup sites. The videos could be useful not only for illiterate subjects but also for literate ones to increase their health awareness.

### Limitations

Because we selected sites around Dhaka in the first year due to a logistic problem, we assessed the 1-year after-effects of the program only around Dhaka. However, because the first checkup results around Dhaka are similar to those of the second year site, except for the urine protein test, we consider that the program has a similar effect even other areas.

### Conclusions

The present study findings suggest that our eHealth system, combining a health checkup and teleconsultation via the mobile network, is an effective tool in the social health care system in developing countries. It also suggests that the stratification rule is working effectively.

In the future, we plan to continue large-scale research into the results of our program, evaluating long-term outcomes to better assess the quality of the service. We will investigate changes in mortality and the frequency of clinic and hospital visits as well as changes in the basic health level and the total costs involved.

## Abbreviations

AUC: area under curve
BAN: body area network
FPR: false positive rate
NCD: non-communicable diseases
PHC: portable health clinic
ROC: receiver operating characteristic
SBP: systolic blood pressure
TPR: true positive rate
